# Development of a Green Downstream Process for the Valorization of *Porphyridium cruentum* Biomass

**DOI:** 10.3390/molecules24081564

**Published:** 2019-04-20

**Authors:** Rocío Gallego, Marina Martínez, Alejandro Cifuentes, Elena Ibáñez, Miguel Herrero

**Affiliations:** Laboratory of Foodomics, Institute of Food Science Research (CIAL, CSIC-UAM), Calle Nicolás Cabrera 9, 28049 Madrid, Spain; rocio.gallego@csic.es (R.G.); marinamtnez95@gmail.com (M.M.); a.cifuentes@csic.es (A.C.); elena.ibanez@csic.es (E.I.)

**Keywords:** pressurized liquid extraction, carotenoids, phycoerythrin, sulfated polysaccharides, microalgae, *Porphyridium cruentum*, biorefinery

## Abstract

As the interest in biorefinery approaches is continuously increasing, new alternatives for the downstream valorization of biomasses are sought. *Porphyridium cruentum* microalga is a good natural source for a variety of interesting bioactive compounds, including carotenoids, phycoerythrin, and sulfated polysaccharides. In the present contribution, the use of compressed fluids-based techniques is explored towards the efficient and green extraction of bioactive compounds to valorize microalgal biomass. The extraction of carotenoids was first optimized using pressurized ethanol. The best extraction conditions involved the use of 125 °C for 20 min at 10.5 MPa. Subsequently, a sequential valorization process was devised based on the application of different steps directed towards the extraction of phycoerythrin, sulfated polysaccharides, and carotenoids, respectively. The applied pressurized conditions allowed the attainment of a good recovery of polar components without compromising the stability and extraction of carotenoids. Therefore, the proposed approach could be employed to obtain different bioactives from *P. cruentum* microalgal biomass employing green extraction processes.

## 1. Introduction

Microalgae have attracted significant attention in recent times as a potential future new food source, creating a great expectancy to confirm if these microorganisms can be a part of our daily diet. In the meantime, what is already widely accepted is that these organisms are a promising source of high added-value products [1]. There is an extensive variety of strains and species that are commercially used to obtain a particular compound of interest. A notable example is the use of *Dunaliella salina* to produce β-carotene [2]. One of the most important features of microalgae is that they may overproduce and accumulate a particular metabolite under specific growing conditions. Thus, they might function as bioreactors. Moreover, other characteristics that make the cultivation of microalgae advantageous include that, i) they can be produced in non-arable land, therefore, not competing with other food production, ii) they only need light energy, carbon dioxide, and some other nutrients, and, iii) these organisms have higher photosynthetic efficiency than plants for the production of biomass [3]. This latter point implies that biomass production is generally faster. 

Considering all these characteristics, microalgae have been proposed as perfect candidates to be included in biorefinery approaches [4]. In a biorefinery, multiple integrated processes are combined for the conversion of biomass into energy and a variety of products, mainly biofuels and added-value products, in a sustainable approach. These aspects are in line with the main features of circular economy, fostered by the European Union, as well as other international organizations, in which exhaustive recycling and re-use in every step of the production chain is sought [5]. In this regard, the compounds obtained from microalgae biomass have the potential to be further employed as high value products in the food and cosmetic industries, among others. For this reason, the development of downstream processes for the extraction and fractionation of those compounds is of special interest. Those processes should be able to be coupled and integrated with sustainable biomass production procedures. Moreover, it is preferred that they are part of a single platform able to extract and fractionate the most interesting compounds efficiently, maintain a high degree of sustainability, and fulfill green chemistry principles. The development of these kinds of green processes implies, therefore, a number of important challenges.

Compressed fluids-based extraction and fractionation techniques have been already shown as good alternatives to be employed in this context [6,7,8,9]. The use of supercritical fluids extraction (SFE), gas-expanded liquids extraction (GXL), and pressurized liquids extraction (PLE) always using green and safe solvents, including water, ethanol or CO_2_, can be effective for the extraction of different components, in agreement with their polarity [7]. Particularly, the use of PLE provides some advantages, such as the attainment of high extraction yields in shorter processing times and with less solvent consumption compared to conventional extraction techniques. The use of solvents at high temperatures and pressures while maintaining their liquid state during the whole extraction procedure is responsible for these interesting features [9]. PLE has been studied for the extraction of high added-value products from microalgae, such as different carotenoids [10], fatty acids and other lipids [11], as well as other bioactive compounds [12,13].

*Porphyridium cruentum* is a red microalga presenting an interesting array of potentially high added-value constituents, although growing conditions have a direct influence on the precise chemical composition of the resulting biomass [14]. Among the bioactive components in *P. cruentum*, carotenoids, mainly zeaxanthin, are highlighted [15,16,17]. Apart from their well-known antioxidant activity, carotenoids are considered active metabolites that may exert protective effects against degenerative diseases, cancer or even type 2 diabetes [18]. Moreover, they can also have a skin protective action [19] and, thus, are highly valued in the cosmetic industry. Moreover, *P. cruentum* contains a high amount of other lipids, mainly characterized by the presence of unsaturated fatty acids, proteins [15], and sulfated exopolysaccharides that constitute their cell wall [20]. These latter polysaccharides possess a typical chemical structure able to confer interesting rheological properties [21]. In addition to this technological function, sulfated polysaccharides have been pointed out by exerting different biological activities [22], including immunomodulatory, anti-inflammatory [23], hypocholesterolemic [20], antimicrobial, and antiviral activities [24]. On the other hand, B-phycoerythrin is considered the main protein found in *P. cruentum*; this is a phycobiliprotein that contains a pigment and has a good value as a fluorescent marker in biomedical applications, although some functional properties have also been described [25].

Thus, considering the complex chemical composition of *P. cruentum*, the present research aimed to develop a new downstream processing platform to efficiently extract the target carotenoids from this microalga, also involving the fractionation of the other bioactive constituents. Different solvents and extraction conditions were studied using PLE and different tools were employed to chemically characterize the fractions obtained, mainly HPLC-DAD-MS and in-vitro assays.

## 2. Results and Discussion

With the aim to develop a biorefinery strategy to recover the most interesting compounds from the *Porphyridium cruentum* biomass, a sequential downstream process was implemented considering: (i) the nature of the target bioactive compounds; (ii) the need for fast and efficient extraction processes to fractionate the biomass; (iii) the use of green solvents, and; (iv) the minimization of operational costs by trying to avoid repetitive heating and cooling operations. Taking into account the mentioned considerations, experiments focused on studying the best pressurized liquids extraction conditions to obtain the highest possible amount of carotenoids were performed first.

### 2.1. Optimization of the PLE of Carotenoids from Porphyridium cruentum

In the first stage, *P. cruentum* biomass was evaluated as a potential source of carotenoids (Figure S1), mainly zeaxanthin. It is important to highlight that the exact chemical composition of the biomass may significantly differ depending on the extraction conditions applied, both quali- and quantitatively. In this sense, although some works showed that supercritical CO_2_ could be a good alternative for the extraction of carotenoids from microalgae [7,8], considering that zeaxanthin is a xanthophyll, thus, belonging to the group of the most polar carotenoids, pressurized ethanol was selected for its extraction. Based on our previous experience using PLE, the nature of the extracting solvent together with the extraction temperature are the most influential parameters in this kind of process. The static extraction time was set at 20 min, which has been shown to be appropriate for the extraction of carotenoids by PLE [9]. The extraction pressure was kept at 10.5 MPa, which is sufficient to maintain the ethanol in the liquid state. Under those conditions, five different extraction temperatures were studied, from 50 to 150 °C. The obtained extracts were characterized in terms of extraction yield and total carotenoids. Moreover, a method based on liquid chromatography coupled to diode array and mass spectrometry detection (HPLC-DAD-MS) was employed to quantify the particular amount of zeaxanthin contained. Results from this study are summarized in Table 1. As it can be observed, total extraction yields increased with temperature, as expected. High temperature increases solute solubility while it favors a faster mass transfer rate and produces a decrease in solvent viscosity, which results in higher yields. However, the highest extraction yield obtained reached 11.4%, which is comparatively lower than the total extraction yield obtained for other microalgae by using pressurized ethanol, such as *Scenedesmus obliquus* [8], *Chlorella vulgaris* [26] or *Neochloris oleoabundans* [10], ranging from 25 to 35%; even so, it is higher than the extraction yield obtained using conventional extraction with acetone (7.67 ± 0.49%). The amount of total carotenoids found in the PLE extracts was more stable, as it can be observed in Table 1. Independently of the temperature employed, the amount of carotenoids recovered was always higher than using a conventional extraction (13.24 ± 1.86 mg g^−1^ extract). Although no statistically significant differences were observed among the PLE extracts, a clear trend was detected, indicating a better recovery of total carotenoids with the increasing extraction temperature, up to 125 °C. This trend was even more marked in the case of individual amounts of zeaxanthin. Thus, combining the information from the amount of carotenoids present as well as from the total extraction yields, the use of pressurized ethanol at 125 °C was considered as the optimum conditions for further process development.

These extraction conditions based on the use of pressurized ethanol allowed a higher recovery of carotenoids from *P. cruentum* than using supercritical fluids. In fact, previous works reported a recovery of 2.0 ± 0.1 mg g^−1^ extract using supercritical CO_2_ and 5.2 ± 0.7 mg g^−1^ using subcritical *n*-butane [17]. These results are in agreement with the relative polarity of zeaxanthin and other carotenoids present in this biomass for which pressurized ethanol seems to be more suitable. This is further supported comparing our results to those previously obtained using acetone–methanol (70:30 v/v), reaching 167 mg 100 g^−1^ of dry biomass [16]. Using the optimum conditions (PLE, ethanol, 125 °C) and considering the extraction yield produced, 388 mg 100 g^−1^ are attained in the present work.

### 2.2. Optimization of Downstream Valorization Processing

#### 2.2.1. Pressurized Aqueous Extractions (Steps 1 and 2)

**Figure 1:**
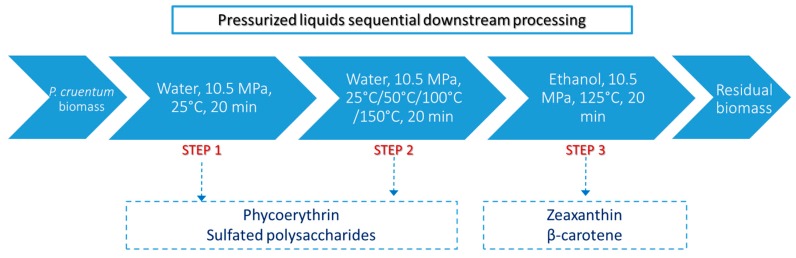
Scheme of the downstream processing proposed for the valorization *of Porphyridium cruentum* biomass based on the use of pressurized liquids.

Considering the particular chemical composition of *P. cruentum*, other compounds, such as proteins and polysaccharides, can also be employed as high added-value products. In this sense, a sequential process was designed taking into account the possibility of extracting phycoerythrin and sulfated exopolysaccharides, together with carotenoids. Although at first sight, the previously optimized PLE conditions for carotenoids could be employed for phycoerythrin extraction, given the thermal lability of phycoerythrin and the need for its recovery as intact phycobiliprotein, a process starting at a lower temperature would be the most appropriate. Thus, the sequential process should unavoidably start with the extraction of phycoerythrin and sulfated polysaccharides and then, finalizing with the extraction of carotenoids. Considering these aspects, the sequential extraction process proposed consisted of three different extraction steps. Its scheme is presented in Figure 1.

The proposed sequential downstream valorization process consists of a three-step extraction protocol to recover three different and unique fractions containing high added-value compounds. Step 1 was primarily directed towards the extraction of phycoerythrin. Considering the nature of this protein, a first extraction with pressurized water at 25 °C was selected. This decision was based on the use of water at low temperature as a usual medium for phycoerythrin extraction [27]. In the second step, the extraction of polysaccharides was targeted. For this step, different extraction temperatures were tested from 25 to 150 °C (Figure 1) with the aim to increase the extraction of polysaccharides while producing a selective extraction between proteins and polysaccharides, both soluble in water. For the third and final step, the extraction conditions previously optimized for the extraction of carotenoids were applied. Results corresponding to the pressurized aqueous extractions are summarized in Table 2. As can be observed, the extraction carried out in the first step is able to recover both phycoerythrin and sulfated polysaccharides, although it does not produce the complete extraction of these components. In fact, during the second step, further extraction of both is obtained, regardless of the extraction temperature selected. As the extraction temperature increases in the second step, the extraction yield is improved. However, total phycoerythrin is best extracted at 25 °C, and higher temperatures resulted in lower extracted amounts, most probably due to thermal degradation. In the case of carbohydrates, the application of 150 °C produced the highest extraction of both total carbohydrates and sulfated polysaccharides, although not statistically significant differences were observed from 50 to 150 °C. It is worth mentioning that even using 25 °C, thus, maintaining the same extraction temperature, the recovery of both groups of components was improved compared to step 1, probably due to the weakening of the cell structure produced by pressure in each extraction cycle.

It is interesting to remark that these processes were not aimed at the recovery of pure phycoerythrin or polysaccharides, as further processing is needed to produce pure compounds [14,27], but to obtain valuable fractions that can be effectively used in other fields, such as the cosmetic industry, due to their interesting composition [14]. In this regard, the use of 50 °C in the second step allows a slight increase in total extraction yield, at the cost of less pure extracts (less concentration in the extract). In this sense, the obtained extracts could be either used independently or combined to have a greater extent of bioactive compounds. Following these extractions, step 3 was carried out to study how the extraction conditions used in steps 1 and 2 could influence the extraction of carotenoids.

#### 2.2.2. Pressurized Ethanol Extraction (Step 3)

Table 3 shows the results obtained in terms of total carotenoids, as well as the individual quantified amounts of zeaxanthin and β-carotene present in the extracts produced during step 3 using the previously optimized extraction conditions (ethanol, 125 °C, 20 min) after each different extraction conditions used in step 2. As can be observed, extraction yield was not significantly affected by the previous extraction protocols independently of the used temperature in step 2. However, the situation was not the same in the case of total carotenoids. Indeed, extraction temperatures higher than 50 °C in step 2, negatively affected the recovery of carotenoids in step 3. This could be explained by a combination of the effect of different pressurization–depressurization cycles, as well as the extraction temperature itself. However, when zeaxanthin and β-carotene are considered individually (quantified by HPLC-DAD), the extraction temperature in step 2 could be increased to 100 °C without producing a significant decrease in their recovery.

#### 2.2.3. Selection of Final Downstream Processing Conditions and Characterization of Extracts

**Figure 2:**
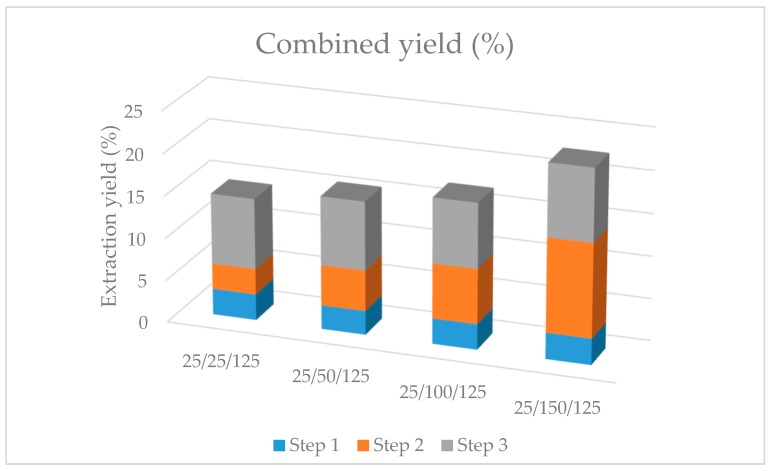
Accumulated extraction yields produced in each sequential processing alternative at the indicated extraction temperatures (°C, step 1/step 2/step 3).

**Figure 3:**
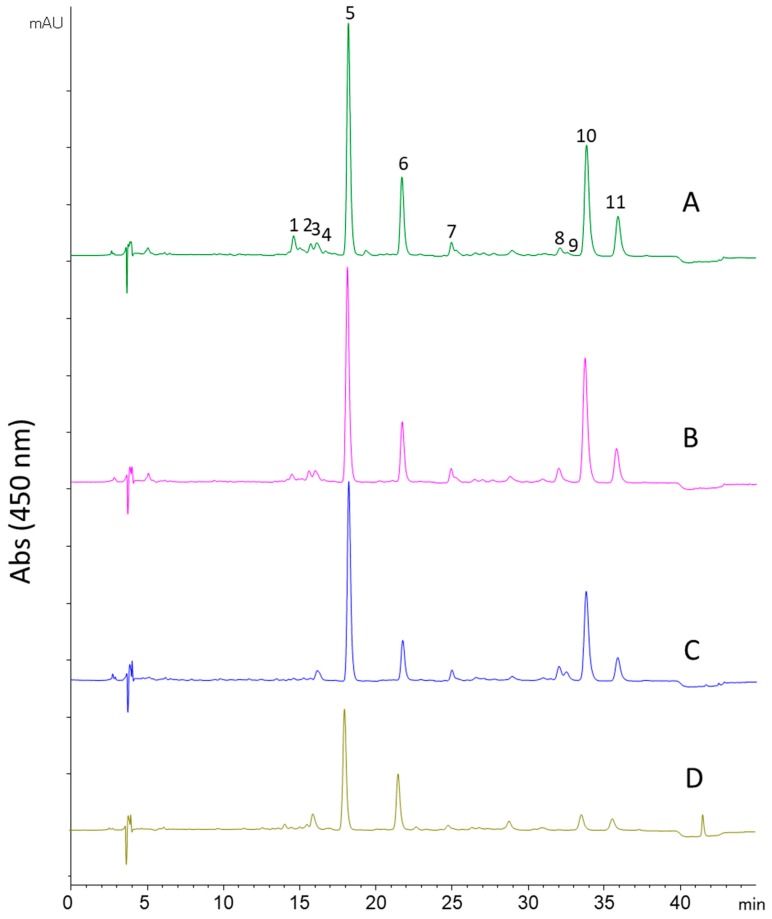
Chromatograms obtained by high-performance liquid chromatography (450 nm) for the extracts obtained in step 3 using ethanol at 125 °C after the extraction in step 2 at (**A**) 25, (**B**) 50, (**C**) 100, and (**D**) 150 °C. For peak assignment, see Table 4.

Different interpretations could be drawn from the results obtained in the optimization of the sequential process. In terms of total extraction yield and, thus, biomass consumption, the use of the highest possible extraction temperatures in each step was clearly more favorable, as can be appreciated from Figure 2. However, the application of those conditions (25 °C, step 1 + 150 °C, step 2 + 125 °C, step 3) would imply a significant decrease on the total amount of carotenoids recovered in the last step (Table 3). This is also easily observed in Figure 3, where chromatograms obtained from the different step 3 extracts are analyzed by HPLC-DAD-MS. As can be seen, the profile obtained in step 3 for all the sequential processes was very similar, although, in agreement with the quantitative data already presented (Table 3), the amount of carotenoids was markedly decreased when the temperature in step 2 was above 50 °C (Figure 3C,D). The main carotenoid was zeaxanthin in all cases, followed by β-carotene. Other carotenoids were detected based on their typical UV–Vis spectra, although no complete assignment was possible. Moreover, other peaks corresponding to chlorophyll a and chlorophyll derivatives were also detected, whose presence has been already reported in red microalgae [14]. The information collected in these analyses is summarized in Table 4, including UV–Vis maxima, molecular ions, and main MS/MS fragments if produced.

When seeking the maximization of the presence of the bioactive compounds in the extracts, the most suitable selection would be to use the lowest possible extraction temperature in step 2. The use of two cycles at 25 °C (steps 1 and 2), followed by step 3 would allow the recovery of 26.6 mg g^−1^ extract of phycoerythrin and at the same time, a total of 19.1 mg g^−1^ extract of sulfated polysaccharides in the water extract and 41.53 mg g^−1^ extract of total carotenoids in the ethanolic extract. This way, two different streams are produced from the biomass, one rich in water-soluble bioactive compounds and a second enriched in carotenoids. Higher combined total amounts of phycoerythrin and sulfated polysaccharides would be attainable if step 2 was carried out at 50 °C thanks to the increase in the extraction yield produced and without affecting the recovery of carotenoids in step 3. In this latter process, however, less pure extracts on the target components would be obtained from step 2. Concerning the amount of carotenoids recovered using the proposed sequential process, up to 118 mg 100 g^−1^ dry biomass and 54 mg 100 g^−1^ dry biomass of zeaxanthin and β-carotene were reached (considering the total extraction yield produced in step 3). These data favorably compares to other extractions reported in the literature using 90% aqueous acetone that obtained 27 mg 100 g^−1^ dry biomass and 24 mg 100 g^−1^ dry biomass of zeaxanthin and β-carotene, respectively, from *P. purpureum* [28].

Additional work is necessary to further process the residual biomass obtained after extraction to complete the valorization sequence.

## 3. Materials and Methods

### 3.1. Samples and Reagents

HPLC-grade solvents including methyl *tert*-butyl ether (MTBE), methanol, acetone, ethanol, and sulfuric acid were purchased from VWR (Leuven, Belgium). Sea sand (0.25–0.30 mm diameter) was from Panreac (Castellar del Vallés, Spain). Butylated hydroxytoluene (BHT), phenol, toluidine blue and standards of d-(+)-glucose, and β-carotene (from *Anacystis nidulans* algae), were obtained from Sigma-Aldrich (St Louis, MO, USA), whereas zeaxanthin and fucoidan (from *Fucus serratus* algae) were acquired from Carbosynth Limited, Berkshire, UK). The water used was Milli-Q water (Millipore, Billerica, MA, USA). The marine Rhodophyta *Porphyridium cruentum* microalgae, consisting of a freeze-dry powder, were kindly donated by Microphyt (Baillargues, France) and stored at 4 °C until use. Briefly, the following growing conditions were applied for biomass production: *P. cruentum* was grown in 5000 L photobioreactor (PBR) consisting in 1.2 km of glass tubes, each, with co-circulation of liquid medium and CO_2_ enriched air. The PBR was under a greenhouse allowing the control of temperature (between 22 and 28 °C) and the intensity of natural light with curtains. The pH was set at 7.5 and was automatically controlled by CO_2_ injection monitored by an inline pH probe (Fermprobe F-235, Broadley James, Silsoe, UK). Air was injected continuously at a rate of 35 L min^−1^. The culture medium used was a marine type medium, corresponding to a modified Hemerick’s medium by the addition of N and P up to 20 and 4 mmol L^−1^, respectively. Cultivation was conducted in a semi-continuous condition enabling to maintain the exponential growth phase. Cells were harvested by bowl centrifugation at 6000 rpm (KG 8006, GEA, Oelde, Germany) and concentrated to around 10 to 13% of dry matter, frozen at −20 °C in polyethylene bags, heat sealed and stored at −20 °C before freeze drying.

### 3.2. Pressurized Liquid Extraction

#### 3.2.1. Equipment

All extractions of *P. cruentum* were carried out in an accelerated solvent extraction system (ASE 200, Dionex, Sunnyvale, CA, USA) equipped with a solvent controller.

Extractions were performed using ethanol or water as solvents at different extraction temperatures, according to the biorefinery design. Extraction time was set at 20 min. Before each extraction, an extraction cell heat-up step was carried out for a given time, fixed by the system (i.e., 5 min when the extraction temperature was 40 and 100 °C, and 8 min at 170 °C). Extractions were performed using 11-mL extraction cells at 10.5 MPa. Depending on the extraction solvent, the introduction of the sample within the extraction cell was different. During steps involving water as a solvent, a small package made of filter paper was introduced into the extraction cells and filled with 1 g of dry algae (or remaining residues after first aqueous extraction). After these extractions, each residue was recovered, lyophilized and used as raw material for the last extraction step. Thus, when ethanol was used as extraction solvent, 1 g of dry microalgae (or remaining residues after the second aqueous extraction) were sandwiched between sand layers (approximately 2 g). All experiments were, at least, duplicated. The extracts obtained were protected from light and frozen. Water extracts were lyophilized in a freeze-drier (Lyobeta, Telstar, Terrassa, Spain), while ethanolic extracts were evaporated under nitrogen stream. Samples were stored at −20 °C to prevent degradation until analysis.

#### 3.2.2. Optimization of the Carotenoids Extraction from *P. cruentum*

First, an optimization of the ethanolic extraction was performed to recover carotenoids. The studied variable was the extraction temperature (50–150 °C), whereas extraction pressure and time were fixed (10.5 MPa and 20 min, respectively). Extraction yield (% dry weight of extract/dry weight of initial biomass), total carotenoids and independent carotenoids (zeaxanthin and β-carotene) were quantified to choose the optimum conditions.

#### 3.2.3. Sequential Process for the Downstream Valorization of *P. cruentum*

A sequential process was developed for the valorization of bioactives from *P. cruentum*. Figure 1 shows a scheme of the entire optimized process. After obtaining the optimum conditions for the recovery of carotenoids, the integrated process was performed in three sequential steps using (1) pure water at 25 °C, (2) pure water at different temperatures (25–150 °C), and (3) pure ethanol at the optimum temperature determined in the optimization phase described in Section 3.2.2. This way, three valuable isolated fractions were obtained from the microalgae biomass.

Step 1: The first step was focused on the recovery of phycoerythrin, which is known to be very sensitive to temperature. For this reason, this step was carried out at 25 °C using water as an extraction solvent and the same conditions of extraction pressure and time (10.5 MPa and 20 min).

Step 2: The residual biomass from the previous extraction was extracted again using PLE at 10.5 MPa for 20 min, using pure water. In this step, four extraction temperatures were studied (25–150 °C) with the goal to optimize the extraction of total and sulfated polysaccharides.

Step 3: In the third and last step, the extraction was performed at the optimum extraction conditions, as described in Section 3.2.2., using the same extraction pressure and static time as all previous extractions.

#### 3.2.4. Conventional Extraction Methods

Conventional acetone extraction was carried out (in duplicate) to determine the total extractable carotenoids in *P. cruentum* using a method proposed elsewhere [6,7] as a benchmark method. Briefly, 200 mg of lyophilized algae was mixed with 20 mL acetone containing 0.1% (w/v) BHT and the mixture was shaken for 24 h under agitation (at 250 rpm in the dark). After centrifugation (10,000 rpm at 4 °C for 10 min), the supernatant was collected, and N_2_ stream was used to remove the solvent. Dry acetone and aqueous extracts were weighted and stored at −20 °C.

### 3.3. Total Carotenoids Determination

A simple spectrophotometric method was used to determine the total carotenoids and total chlorophylls concentration, based on their characteristic absorbance, as described elsewhere [7]. Extracts obtained during optimization and from step 3 were dissolved in methanol at a concentration of 0.3 mg mL^−1^. The absorbance of these solutions was recorded at a specific wavelength (470 nm) for total carotenoids. An external standard calibration curve of zeaxanthin (0.2–20 μg mL^−1^) was used to calculate the total carotenoids content. Total carotenoids were expressed as milligram carotenoids per gram extract.

### 3.4. Identification and Quantification of Carotenoids by Liquid Chromatography Coupled to Mass Spectrometry

The profile of carotenoids of *P. cruentum* ethanolic extracts (optimization and steps 1–3) was determined by HPLC-DAD-MS/MS, according to a method previously described [7,8,10], with some modifications. HPLC analyses of the extracts were carried out in an Agilent 1100 series liquid chromatograph (Santa Clara, CA, USA), equipped with a diode-array detector (DAD) and using a YMC-C30 reversed-phase column (250 mm × 4.6 mm i.d., 5 μm particle size; YMC Europe, Schermbeck, Germany) and a pre-column YMC-C30 (10 mm × 4 mmi.d., 5 μm). The mobile phases were mixtures of methanol–MTBE–water (90:7:3 v/v/v) (solvent A) and methanol–MTBE (10:90 v/v) (solvent B). Carotenoids were eluted according to the following gradient: 0 min, 0% B; 20 min, 30% B; 35 min, 40% B; 38 min, 80% B; 43 min, 100% B; 45 min, 0% B. The injection volume was 10 μL, whereas the flow rate was 0.8 mL min^−1^. The detection was performed at 280, 450, and 660 nm, although spectra from 240 to 770 nm were recorded using the DAD (peak width 0.1 min (2 s), slit 4 nm). The instrument was controlled by LC ChemStation 3D Software Rev. B.04.03 (Agilent Technologies, Santa Clara, CA, USA). Extracts were dissolved in pure ethanol at an appropriate concentration (1–10 mg mL^−1^) and filtered through 0.45 μm nylon filters before HPLC analysis. For the calibration curves, five different concentrations of zeaxanthin in ethanol, (from 3.9 to 62.5 μg mL^−1^) and seven concentrations of β-carotene in ethanol (from 31.3 to 1000 μg mL^−1^) were analyzed, at least, by duplicate using the high performance liquid chromatography coupled to diode array detection (HPLC-DAD) instrument for the calibration curve.

The same instrument was directly coupled at the exit of the DAD to an ion trap mass spectrometer (Agilent ion trap 6320, Agilent Technologies, Santa Clara, CA, USA) via an atmospheric pressure chemical ionization (APCI) interface. Analyses were conducted under positive ionization mode using the following parameters: capillary voltage, −3.5 kV; drying temperature, 350 °C; vaporizer temperature, 400 °C; drying gas flow rate, 5 L min^−1^; nebulizer gas pressure, 60 psi; corona current (which sets the discharge amperage for the APCI source), 4000 nA. Full scan spectrum was acquired in the range from *m/z* 150 to 1300. Automatic tandem mass spectrometry (MS/MS) analyses were also performed, fragmenting the two highest precursor ions (10,000 counts threshold; 1 V Fragmentor amplitude).

### 3.5. Determination of B-Phycoerythrin

The amount of phycoerythrin (B-PE) in the extracts of *P. cruentum* was calculated using a spectrophotometric method proposed by Bermejo Román et al. [29]. Extracts were diluted in Milli-Q water at 1 mg mL^−1^ and absorbances were measured at 565, 620, and 650 nm. B-PE content (expressed in mg mL^−1^) was determined using the following equations.

R-phycocyanin [R-PC] = (Abs 620 − 0.7 × Abs 650)/7.38(1)

Allophycocyanin: [APC] = (Abs 650 − 0.19 × Abs 620)/5.65(2)

B-Phycoerythrin: [B-PE] = (Abs 565 − 2.8 × [R-PC] − 1.34 × [APC])/12.27(3)

### 3.6. Determination of Carbohydrates and Sulfated Polysaccharides

Total carbohydrates content of the extracts of *P. cruentum* obtained using water as solvent was determined using the phenol-sulfuric acid method, following the colorimetric analysis proposed by Geresh et al. [30], with some modifications. Briefly, 167 μL 5% phenol was mixed with 278 μL of extract (diluted in Milli-Q water at known concentration). Then, 1000 μL sulfuric acid was added and carefully mixed. After 30 min at room temperature, 300 μL of the mixture was transferred into a 96-well microplate and absorbance was recorded at 490 nm. A standard calibration curve using glucose (0.03–1.0 mg mL^−1^) was used to determine the carbohydrates content, which was expressed as milligram total carbohydrates per gram extract.

Spectrophotometric analysis was carried out to determine sulfated polysaccharides of aqueous extracts of *P. cruentum*, following the method proposed by Esteves et al. [31]. Briefly, 1 mL toluidine blue solution (at 0.01 mg mL^−1^) was added to 200 μL of extract (diluted in Milli-Q water at 10 mg mL^−1^). After 10 min in darkness, 300 μL of the mixture was transferred into a 96-well microplate and absorbance was recorded at 620 nm. Fucoidan was used for the calibration curve, from 3.9 to 125 μg mL^−1^ and results were expressed as milligram sulfated polysaccharides per gram extract.

### 3.7. Statistical Analyses

IBM SPSS Statistics software v.19 was employed for data elaboration and statistical analysis using a level of significance set at 95%. One-way analysis of variance (ANOVA), together with Student–Newman–Keuls test were employed to group extracts, based on statistically significant differences. Mean values were compared using the Tukey’s test, and the differences were considered statistically significant if *p* < 0.05.

## 4. Conclusions

This work describes the optimization of a downstream compressed fluids-based process to recover bioactive compounds from *Porphyridium cruentum* biomass for the first time. Different pressurized solvents and conditions were applied to sequentially extract phycoerythrin, sulfated polysaccharides, and carotenoids from this microalga. The obtained results showed that low extraction temperatures using pressurized water were necessary to extract phycoerythrin and sulfated polysaccharides. Temperatures higher than 50 °C produced a decrease in the amount of both groups of compounds and, more importantly, negatively affected the subsequent extraction of carotenoids. As carotenoids were the main target of the present work, the optimization of the extraction conditions meant that 125 °C and 20 min were the most suitable values to maximize their recovery using pressurized ethanol. This way, polar components were extracted in two first consecutive steps, whereas carotenoids were obtained in a third step. The process could be scalable although further work could be needed to produce a full valorization of the algal biomass.

## Figures and Tables

**Table 1:** Extraction yield, total carotenoids and zeaxanthin amounts (mg g^−1^ extract) determined in the extracts obtained using pressurized ethanol at the indicated extraction temperature. Other extraction conditions: extraction pressure, 10.5 MPa; extraction time, 20 min.

Extraction Temperature (°C)	Extraction Yield (%)	Total Carotenoids (mg g^−1^ Extract)	Zeaxanthin (mg g^−1^ Extract)
50	3.12 ± 0.21 ^a^	35.41 ± 0.15	12.29 ± 2.13 ^a^
75	4.13 ± 0.16 ^a^	36.09 ± 3.95	13.85 ± 0.76 ^a^
100	6.42 ± 0.62 ^b^	39.11 ± 1.96	12.48 ± 2.89 ^a^
125	9.00 ± 0.67 ^c^	43.15 ± 0.84	19.11 ± 4.33 ^a^
150	11.36 ± 0.11 ^d^	38.22 ± 3.14	4.87 ± 0.18 ^b^

Note: Each data point represents the mean ± SD of replicates. Different superscript letters indicate statistically significant differences (*p* < 0.05).

**Table 2:** Extraction yield, total phycoerythrin (mg g^−1^ extract), total carbohydrates (mg g^−1^ extract) and sulfated polysaccharides amounts (mg g^−1^ extract) determined in the extracts obtained using pressurized water at the indicated conditions during steps 1 and 2. Other extraction conditions: extraction pressure, 10.5 MPa; extraction time, 20 min.

Extraction Temperature (°C)	Extraction Yield (%)	Total Phycoerythrin (mg g^−1^ Extract)	Total Carbohydrates (mg g^−1^ Extract)	Sulfated Polysaccharides (mg g^−1^ Extract)
*Step 1*				
25	3.03 ± 0.19 ^a^	13.21 ± 0.77 ^a^	83.57 ± 11.78 ^a^	8.88 ± 0.85 ^a^
*Step 2*				
25	3.06 ± 0.06 ^a^	25.02 ± 2.89 ^b^	97.17 ± 6.65 ^a,b^	10.22 ± 0.14 ^a,b^
50	4.81 ± 0.04 ^b^	16.51 ± 0.21 ^b^	106.34 ± 11.63 ^a,b,c^	9.80 ± 0.21 ^a,b^
100	6.51 ± 0.20 ^c^	5.99 ± 0.35 ^a,c^	108.53 ± 4.47 ^a,b,c^	9.94 ± 0.24 ^a,b^
150	11.12 ± 0.26 ^d^	4.28 ± 0.10 ^c^	122.97 ± 5.61 ^c^	11.47 ± 0.74 ^b^

Note: Each data point represents the mean ± SD of replicates. Different superscript letters indicate statistically significant differences (*p* < 0.05).

**Table 3:** Extraction yield, total carotenoids (mg g^−1^ extract), and individual amounts of zeaxanthin (mg g^−1^ extract) and β-carotene (mg g^−1^ extract) determined in the extracts obtained using pressurized ethanol at 125 °C during step 3 after the indicated conditions during step 2. Other extraction conditions: extraction pressure, 10.5 MPa; extraction time, 20 min.

Extraction Temperature (°C) in Step 2	Extraction Yield (%)	Total Carotenoids (mg g^−1^ Extract)	Zeaxanthin (mg g^−1^ Extract)	β-carotene (mg g^−1^ Extract)
25	8.34 ± 0.08 ^a,b^	41.53 ± 1.74 ^a^	14.09 ± 0.30 ^a^	6.53 ± 0.25 ^a^
50	8.12 ± 0.88 ^a,b^	40.71 ± 1.79 ^a^	13.14 ± 0.21 ^a,b^	7.71 ± 0.93 ^a^
100	7.70 ± 0.51 ^b^	29.43 ± 2.20 ^b^	13.71 ± 2.74 ^a,b^	6.51 ± 1.55 ^a^
150	8.59 ± 0.40 ^a,b^	28.97 ± 0.25 ^b^	6.67 ± 0.66 ^b^	1.55 ± 0.42 ^b^

Note: Each data point represents the mean ± SD of replicates. Different superscript letters indicate statistically significant differences (*p* < 0.05).

**Table 4:** Peak assignment, UV–Vis maxima and mass spectrometry spectra features of the separated compounds contained in the pressurized liquids extraction (PLE) extracts obtained in step 3 using ethanol at 125 °C and 20 min.

Peak #	Identification	t_R_ (min)	UV–Vis Maxima (nm)	[M + H]^+^ *m/z*	Main Fragments Observed (MS/MS)
1	Chlorophyll-derivative	14.63	426, 663	616.2	
2	Chlorophyll-derivative	15.02	425, 663	647.7	
3	Chlorophyll-derivative	15.75	434, 670	664.0	
4	Chlorophyll-derivative	16.09	438, 670	617.8	313.9, 288.0
5	Zeaxanthin *	18.07	420s, 450, 476	569.5	551.9
6	Zeaxanthin isomer	21.42	422s, 445, 472	579.8	
7	Chlorophyll-derivative	24.86	450, 478, 674	718.6	593.5, 453.4
8	Pheophytin a’	31.30	373s, 409, 666	872.3	594.7
9	Pheophytin b	31.78	373s, 409, 666	885.5	
10	β-carotene *	33.31	430s, 450, 475	537.5	
11	Carotenoid	35.28	422s, 446, 472	665.9	

* Identified with a commercial standard; s, spectral shoulder.

## Supplementary Materials

The following are available online, Figure S1.
