# Supplementary material for: Development of a Green Downstream Process for the Valorization of Porphyridium cruentum Biomass
Source: Molecules. 2019 Apr 20;24(8):1564. doi: 10.3390/molecules24081564 (PMC6515528; doi:10.3390/molecules24081564)
Supplement: Supplementary file 1 [file molecules-24-01564-s001.pdf]

## ELECTRONIC SUPPLEMENTARY MATERIAL TO:

# Development of a green downstream process for the valorization of *Porphyridium cruentum* biomass.

Rocío Gallego, Marina Martínez, A. Cifuentes, Elena Ibáñez, Miguel Herrero*

Laboratory of Foodomics, Institute of Food Science Research (CIAL, CSIC-UAM), Calle Nicolás Cabrera 9, 28049 – Madrid, Spain

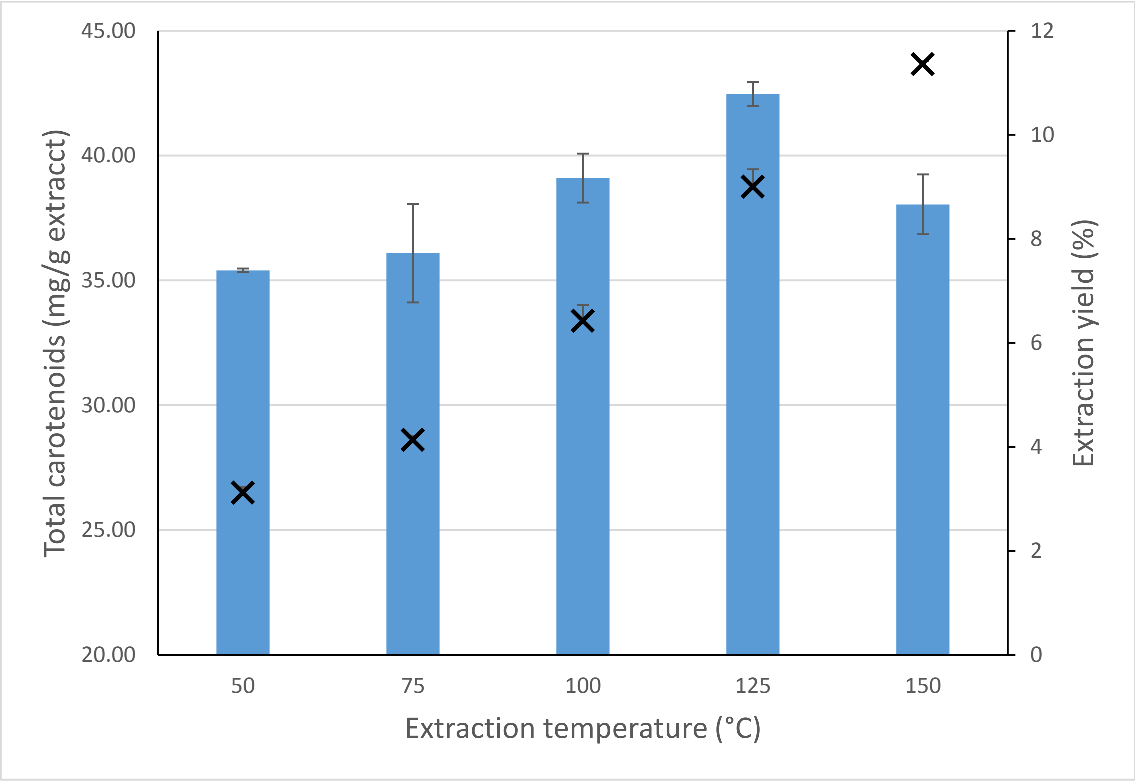


**Figure S1.** Total carotenoids amounts (bars) and extraction yield (x) obtained after the pressurized ethanol extraction of *Porphyridium cruentum* biomass at the indicated extraction temperatures.
